# Deep-Dwelling Populations of Mediterranean *Corallium rubrum* and *Eunicella cavolini*: Distribution, Demography, and Co-Occurrence

**DOI:** 10.3390/biology11020333

**Published:** 2022-02-20

**Authors:** Laura Carugati, Davide Moccia, Lorenzo Bramanti, Rita Cannas, Maria Cristina Follesa, Susanna Salvadori, Alessandro Cau

**Affiliations:** 1 Department of Life and Environmental Sciences, University of Cagliari, Via T. Fiorelli 1, 09126 Cagliari, Italy; mocciadavide@unica.it (D.M.); rcannas@unica.it (R.C.); follesac@unica.it (M.C.F.); salvador@unica.it (S.S.); alessandrocau@unica.it (A.C.); 2 Laboratoire d’Ecogeochimie des Environnements Benthiques (LECOB), Sorbonne Universites, Université Pierre-et-Marie-Curie, Centre National de la Recherche Scientifique (CNRS), Observatoire Oceanologique, F-66650 Banyuls sur Mer, France; lorenzo.bramanti@obs-banyuls.fr

**Keywords:** *Corallium rubrum*, *Eunicella cavolini*, demography, co-occurrence, deep sea, Mediterranean Sea

## Abstract

**Simple Summary:**

Octocorals are marine ecosystem engineers that can form dense patches on rocky substrates. These organisms enhance the three-dimensional complexity of the habitat and provide several ecological services within and around their canopy. Mapping their distribution and understanding the intra- and inter-specific processes that drive population dynamics is of crucial importance. In this study, we investigated via Remotely Operated Vehicle the distribution and demography of two hard-bottom octocorals that share similar habitat preferences within the Western Mediterranean: the yellow gorgonian *Eunicella cavolini* and the precious red coral *Corallium rubrum*. We tested for possible mutual influences of the two species when co-occurring. Here, we show that for both species, populations dwelling in deeper habitats show demographic features of immature or disturbed populations, possibly due to environmental and/or anthropogenic disturbances that occur locally. Moreover, the density of one species is significantly positively correlated to that of the other, but not influenced by the colony morphology (e.g., height) of the other species. We encourage further studies that might contribute to shedding light on inter-specific relations occurring in these vulnerable ecosystems.

**Abstract:**

*Corallium rubrum* and *Eunicella cavolini* are two octocorals, reported as co-occurring species in the deep rocky habitats of the Mediterranean Sea with a high hydrodynamic and moderate eutrophication. Their spatial distribution and demography in the deep sea are mainly affected by temperature and direct and indirect anthropogenic activities; however, knowledge of the factors that potentially influence their co-existence is scarce. This paper provides novel data on the distribution and demography of these two species, at depths between 50 and 290 m in the Western Mediterranean Sea, providing insights on their co-occurrence. Both species exhibited the highest population density at deeper sites (>150 m), showing an inverse size–density relation. Density values ranged from 0.03 colonies m^−2^ to 32 and 80 col. m^−2^ for yellow gorgonian and red coral, respectively. The two species co-occurred in 13% of the total frames examined, mostly dwelling between 120 and 160 m depth. Distance-based linear modeling (DistLM) emphasized that when co-occurring the variability of the two species’ densities were significantly driven by the density—rather than the morphology (i.e., height)—of the other species. We stress the need for further studies to elucidate the possible mutual effects of suspension feeders and to test the role of different environmental factors potentially influencing inter-specific relationships.

## 1. Introduction

Deep-dwelling octocorals often dominate hard-bottom benthic ecosystems, where they act as ecosystem engineers and foundation species, providing three-dimensional habitats for fish and invertebrates, and also contributing to the maintenance of ecosystem functioning [1,2]. They facilitate the presence of associated fauna [3] by being a suitable habitat and refuge for numerous species [4], thus increasing biodiversity. They also enhance the pelagic–benthic transfer of matter and energy by capturing suspended particulate organic matter and plankton [5,6].

The spatial distribution of benthic suspension feeders depends on settlement processes, which can influence feeding success and thus the competition with other corals dwelling within the same community [7]. In general, the over-specialization of food sources at small spatial scale, especially in highly selective environments, could increase the risk of a species being replaced by a more efficient one that targets the same resource, and could also favor the formation of monospecific patches, as in the case of red coral [8].

The highly valuable red coral *Corallium rubrum* (Linnaeus, 1758) is endemic to the Mediterranean Sea and the adjacent Eastern Atlantic Ocean [9], dwelling from 10 to 1000 m depth [10]. Red coral forms arborescent colonies with a lifespan that can exceed 100 years and can reach more than 50 cm in height [11,12]. This species is one of the most precious marine resources and its harvesting dates back to ancient Roman times; indeed, its red calcium carbonate skeleton can be used to produce jewelry and artistic objects. From a management perspective, red coral populations can be distinguished based on their demography and distributions along the bathymetric gradient: (i) shallow-water populations dwelling above 50 m depth, characterized by small-sized and highly dense colonies [13] due to intense harvesting in the last few decades; (ii) deep populations, dwelling between 50 and 130 m depth, composed of large and sparse colonies that are currently the main target of professional harvesting [14,15,16,17]; and (iii) the deepest populations (up to 1000 m), represented by relatively small and sparse colonies [10,18].

Intensive harvesting, along with red coral’s slow growth rate (0.26 mm yr^−1^ for deep-dwelling populations) [19] and sensitivity to environmental stressors, such as positive thermal anomalies [20,21], has increased concerns regarding its fate. Due to commercial and ecological relevance of this species, knowledge on its biological, demographic, and genetic aspects has consistently increased during the last two decades, especially for shallow-water populations [13,16,20,22]. Professional red coral harvesting is performed by scuba divers, who selectively harvest colonies with a basal diameter larger than 7 mm, according to regulations in force in the GFCM competence area. Recent studies have documented how fishing efforts currently reach depths >100 m and shift towards deep-sea populations, which are the least known ones [23]. Thus, there is an urgent need to improve our knowledge on deep-dwelling red coral populations, which differ in terms of density, morphology, reproductive patterns, and genetic structure from their shallower counterparts [14,16,24,25,26].

Despite the frequent reports of monospecific patches of red coral in shallow-water ecosystems, recent studies reported its consistent co-occurrence with the yellow gorgonian *Eunicella cavolini* (Koch, 1887), highlighting a partially overlapping habitat [17,27]. Indeed, both species show an extensive geographic distribution within the Mediterranean basin, a wide bathymetric range, and a preference for rocky bottoms with high hydrodynamic and moderate eutrophication levels [28,29,30]. These features render *E. cavolini* a potential red coral competitor for space and resources [31]. However, to date, no study has provided information on the effects of this potential competition on the distribution and population structures of both species.

*E. cavolini* is a common gorgonian in the Mediterranean Sea, forming distinct facies within coralligenous assemblages [28]. Its distribution area spans from the Western Mediterranean to the Aegean Sea and the Sea of Marmara [28,32], over a wide bathymetric range [28,29]; however, our knowledge regarding its demography is mainly restricted to depths above 50 m [28].

The lack of studies on deeper red coral and yellow gorgonian populations limits our understanding of their ecology and the possibility of developing an effective management plan for their conservation. These species are sensitive to environmental changes [21], and also to the impact of direct (i.e., fishing, contamination) and indirect anthropogenic disturbances (e.g., ocean acidification), which may provoke population fragmentation [17,28,33]. Considering also the genetic separation of deep-dwelling populations from the shallow-water ones, specific measures are urgently needed to guarantee their protection [11]. Effective management actions such as the inclusion of deep-sea banks in marine protected areas (MPAs) may enhance the conservation and recovery of these species, which are crucially important for deep-sea benthic ecosystem functioning.

Remotely Operated Vehicles (ROVs) represent an effective and non-invasive tool, typically used to evaluate the state of the conservation of coral populations dwelling in the bathymetric range of SCUBA-diving operative limits and beyond [17,34].

In this study, we carried out ROV surveys from the northeastern to the southwestern coast of Sardinia (Western Mediterranean Sea) between 50 and 290 m depth in order to: (i) provide additional data on the distribution and population structures of the deep-dwelling populations of *C. rubrum*; (ii) describe the population structures of *E. cavolini* below 50 m depth; and (iii) investigate the patterns of co-occurrence between the two species. All the collected data also constitute a baseline to evaluate the potential impacts of fishing activities and eventual populations recovery.

## 2. Materials and Methods

### 2.1. Study Area

The study area is located around the island of Sardinia (Italy), which is in the central-western region of the Mediterranean Sea. The survey was conducted mostly over the shelf break of the eastern Sardinian continental margin, a passive margin of the Tyrrhenian Basin that is delimited to the north by the “Etruschi” seamount and to the south by the “Ichnusa” seamount. The narrow continental shelf that characterizes the eastern area of the island terminates at about 60–100 m depth in the southern and central areas and at around 200 m depth in the northern area. Along the entire eastern side of the island, the continental shelf and slope are both incised by profound submarine canyons and connected to inland orographic structures and river basins [35,36,37]. Contrarily, western Sardinia is characterized by a wide continental shelf that can extend up to hundreds of kilometers, particularly in the southwestern part of the island. Hydrologically, the whole eastern coast of Sardinia is characterized by the flow of the Levantine Intermediate Water (LIW) throughout its whole extension [38], with a part of the LIW circulating cyclonically in the southern part of the Tyrrhenian Basin and flowing along the northern slope of the Sardinia Channel (SC). The SC is considered one of the crucial areas in the Mediterranean Sea, controlling the exchange of water mass between the eastern and the western basins of the Mediterranean [39].

**Figure 1:**
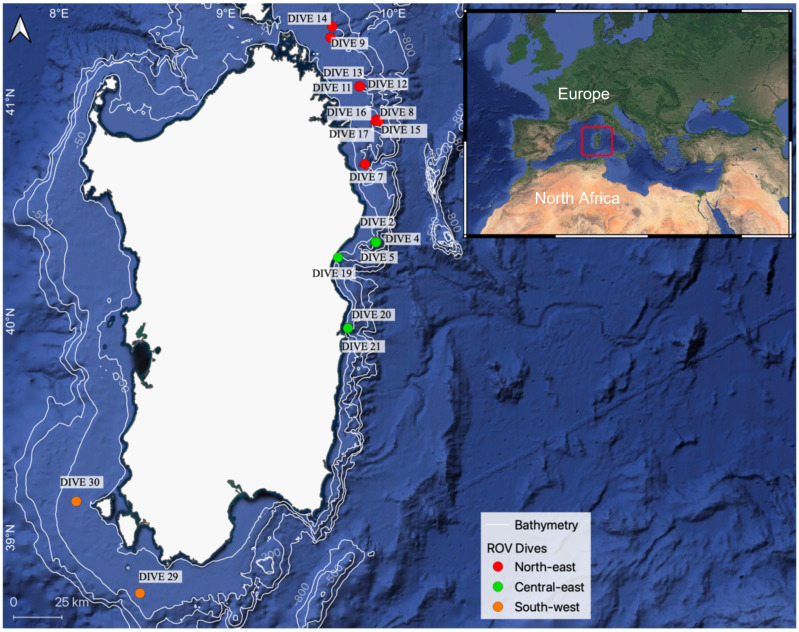
Map of the study area displaying the location of the 19 ROV surveys carried out from the North-east to the South-west Sardinian continental shelf and shelf break.

Overall, nine sites were investigated, which were a priori allocated to three geographical areas, namely: (i) North-east (NE), including Pietrame and the Canyons of Caprera, Mortorio, and Tavolara, with sites located at a greater distance from the nearest coastline (up to 12 Nm from the coast) compared to sites located along the Central-east and South-west coasts (up to 6 and 9 Nm from the coast, respectively); (ii) Central-east (CE), including the Cala Gonone Shoal and the Canyons of Orosei and Arbatax; and (iii) the South-West (SW) area, including Bancotto and Secchitella, which are located over rocky outcrops rising from the wide continental shelf that characterizes the western coast of Sardinia (Figure 1; Table 1).

### 2.2. ROV Surveys—Data Acquisition

ROV surveys were conducted over a depth range between 50 and 290 m depth (Figure 1; Table 1). This study did not involve the sampling or damage of any endangered or protected species and is based on direct observations with ROV footage and image analysis, a non-invasive tool that is particularly suited for high-conservation-interest habitats, such as those under investigation.

The dataset used in this study includes video and photographs collected during a ROV survey carried out in August 2013 onboard the r/v “Astrea”. Each site was explored through a variable number of ROV dives (from a min. of one up to four), to collect video material suitable to perform image analysis.

The ROV “Pollux III” was equipped with a digital camera (Nikon D80, 10 megapixels), a strobe (Nikon SB 400), a high-definition video camera (Sony HDR-HC7), track-link system, depth sensor, compass, and two parallel laser beams providing a constant 10 cm reference scale.

More than 70 h of ROV footage was obtained through 19 dives; footage was recorded moving the ROV at an average speed of ~0.35 knots at ~0.5 meters from the seabed, covering a total investigated surface of more than >4000 m^2^ (Table 1). Transects could not be linear since the survey focused on the target species *C. rubrum*, which shows a patchy distribution over several typologies of hard bottoms, including steep walls, caves, and boulders.

### 2.3. Image Analysis

Frames were randomly extracted from the high-resolution ROV footage using the software “DVDVideosSoft” (www.DVDVideoSoft.com; accessed on 1 December 2021). Overlaying, low-visible, poor-focus, and unsuitable frames (i.e., frames with 100% soft bottom were excluded) were discarded, while keeping only the usable ones for the analysis (see Figure S1 as an example). The analysis of colony density and morphology was performed with CPCe software (Coral Point Count with Excel extensions) [40], using a randomly positioned square of 0.25 m^2^ (i.e., 0.5 × 0.5 m) as a sampling unit in each frame, hereafter referred to as frame [17]. Different patches in the investigated sites were identified. A patch is defined as a group of more than two colonies [41], and a minimum of 10 m in distance was used as a reference to define two distinct patches, with no isolated colonies in between [14].

The substrate composition was estimated using suitable frames and was expressed as the proportion of “hard” (i.e., 100% rocky substrate) and “mixed” bottoms (i.e., hard bottoms with the presence of deposited sand or silt, in variable percentages). We also evaluated the local slope of the substrate (expressed as vertical or horizontal wall) and the presence or absence of deposited sand or silt. Then, we calculated red coral occupancy as the percentage of frames in which red coral was present over the total number of suitable frames in each site. Moreover, the following parameters for *C. rubrum* were retrieved per each frame: (1) density, based on the number of colonies within each frame and expressed as number of colonies m^−2^ [17]; (2) morphological parameters of colonies, such as basal diameter (measured in mm at the inflection point) [19], colony height (measured in cm from the basis to the farthest tips) [42], and the proportion of colonies with a basal diameter larger than 10 mm (Sardinian legal harvesting limit size), and taller than 10 cm [17]; (3) population structure through the analyses of the size-frequency distributions of height and basal diameter, grouping colonies in classes of 2 cm and 2 mm, respectively; (4) orientation of colonies with respect to the substrate, starting from 0° (vertically oriented colonies), 45°, 90°, 135°, up to 180° (colonies in overhanging position) [14]; (5) branching pattern [43], which is a useful ecophenotypic factor and also represents a proxy of the age [44]; and (6) percentage of dead and alive colonies. Similarly, we reported density, colony height, orientation, and the height-frequency distribution for *E. cavolini* (grouping colonies into five classes: 1–10, 11–20, 21–30, 31–40, and >41 cm). In addition, the co-occurrence of red coral colonies with *E. cavolini* in the same frame was also recorded. In particular, we calculated: (i) the proportion of the two species when co-occurring; (ii) demographic (i.e., colony density) and morphological features (i.e., height for both species and diameter only for red coral) when co-occurring and not. Fishing impact was evaluated as the percentage of frames showing the presence of derelict fishing gears, which are fishing devices that are known to cause severe accidental damage to arborescent benthic fauna, such as the species under scrutiny in the present study [45,46]. This indicator was used as a proxy to infer about general fishing activities conducted in the survey area. 

### 2.4. Data Analysis

The descriptive distribution parameters of skewness (g1) and kurtosis (g2) were estimated for the size-frequency distribution per each investigated site. Coefficients of g1 and g2 were considered significant if the ratio to their standard error was >2 [47]. To assess the differences in red coral density, colony height, diameter, maximum order of branches and orientation among areas, between sites within each area, and between colonies co-occurring or not with *E. cavolini*, we used the Permutational Analysis of Variance (PERMANOVA) [48]. The sampling design included three factors as main sources of variance: “area” (fixed; 3 levels), “site” (random, nested in area; 4, 3, and 2 levels for NE, CE, and SW areas, respectively), “co-occurrence” (random, nested in site; 2 levels expressed as presence/absence), using red coral density, colony height, diameter, maximum branching level and orientation separately as response variables. As replicates for the statical analyses, we used density values calculated for each “frame” and each measured value of colony height, basal diameter, maximum branching level, and orientation. The analyses were based on Euclidean distances matrix of no previously transformed data, using 999 permutations of the residuals under a reduced model [49]. For all the analyses, when significant effects of the considered factors were observed, pairwise tests were also carried out. Because of the restricted number of unique permutations, *p* values in the PERMANOVA and pairwise tests were obtained from Monte Carlo samplings [50]. The PERMANOVA analyses were performed using the software PRIMER 7+ [51].

We also aimed to test differences in red coral variables according to water depth, which was pooled in 4 depth categories: 90–110 m, 111–130 m, 131–150 m, and >150 m. Differences in red coral density, height, basal diameter, maximum branching level, and colonies orientation were tested through a Kruskal–Wallis routine, coupled with Mann–Whitney pairwise comparison, using the abovementioned depth categories. The same approach described above was carried out also for *E. cavolini* density, height, and colony orientation.

To identify the potential drivers of the variability of *E. cavolini* and *C. rubrum* density when co-occurred, non-parametric multivariate multiple regression analyses based on Euclidean distances were carried out using the routine DistLM, the forward selection procedure, and adjusted R^2^ and AIC as selection criteria [52]. The forward selection of the predictor variables was carried out with tests by permutation. *p* values were obtained using 999 permutations of the raw data for the marginal tests (test of individual variables), whereas, for all sequential tests, the routine used 999 permutations of residuals. The test was performed on the subset of frames where both species were present (i.e., n = 105; 13% of the analyzed frames). In detail, fourth-root-transformed density values of both *E. cavolini* and *C. rubrum* were used, separately, as response variables. For each of the two response variables, the other species’ density, colony height, and depth were used as potential explanatory covariates.

## 3. Results

### 3.1. Corallium Rubrum: Occupancy, Density, and Population Size Structure

**Figure 2:**
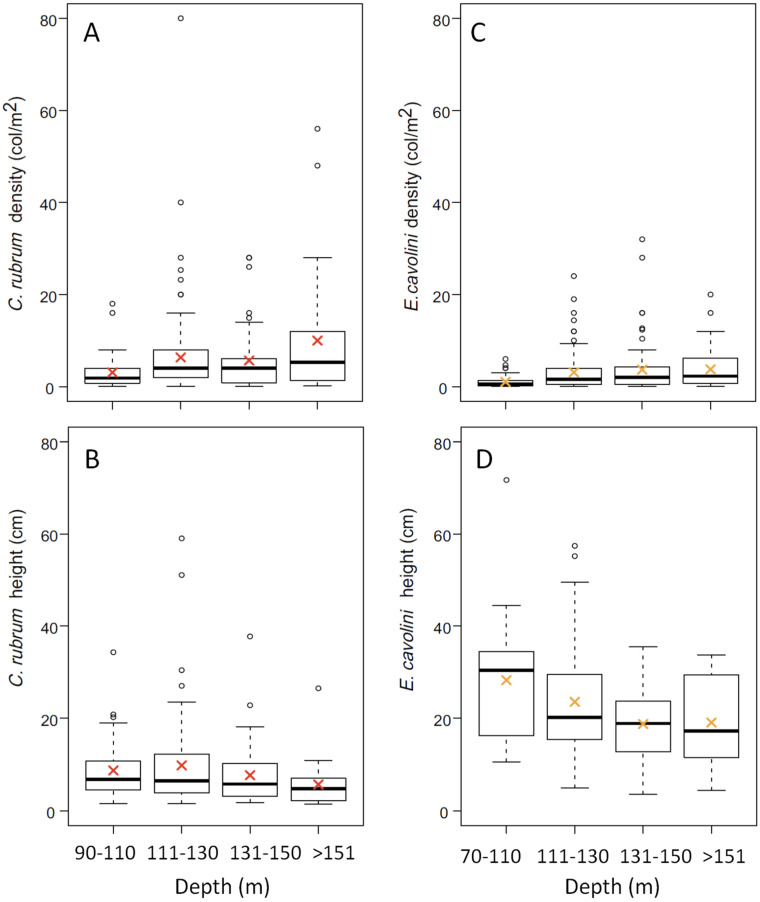
Boxplots showing colony density and height at different depth ranges: (**A**) *C. rubrum* density and (**B**) height; (**C**) *E. cavolini* density and (**D**) height. The thick line shows the median of the data, and the cross indicates the mean value in red for *C. rubrum* and in orange for *E. cavolini*. The small circles are the outliers.

Over a total of 937 frames, 835 were used for the image analysis. Most of the substrate in the total surveyed area (more than 4000 m^2^) consisted of hard bottoms, except for the “Secchitella” site where the substrate was mostly (72%) formed by mixed bottom (hard bottom + sand or mud). In all investigated areas, all patches were settled on vertical and partially silted substrates, with the only exception of six and twelve colonies settled on horizontal substrates in the Mortorio Canyon and Bancotto, respectively. Red coral was found in 249 out of the 835 analyzed frames (30%). A total of 1384 colonies of *C. rubrum* forming 53 red coral patches were counted. Along the NE coast (n = 534 frames), red coral was recorded from a minimum of 22% to a maximum of 44% of frames, with a mean density within patches of 4.5 ± 2.4 colonies m^−2^. In the CE areas (n = 228), the presence of red coral dropped down to 3–33% of frames, with a mean density of 6.3 ± 3.1 colonies m^−2^. Finally, along the SW coast of the island (n = 73), red coral was recorded in 25–37% of the analyzed frames, with the lowest mean density recorded of 2.8 ± 1.4 colonies m^−2^ (Table 2). Red coral density did not significantly change among areas nor among sites within the same area (Table 3). However, it changed significantly along the bathymetric range considered, with higher values of density registered below 110 m depth (Figure 2A; K-W, *p* < 0.01; Supplementary Table S1).

Overall, 449 and 267 colonies in the three studied areas were suitable to be measured for height (NE, n = 334; CE, n = 83; SW, n = 32) and diameter (NE, n = 203; CE, n = 49; SW, n = 15), respectively.

**Figure 3:**
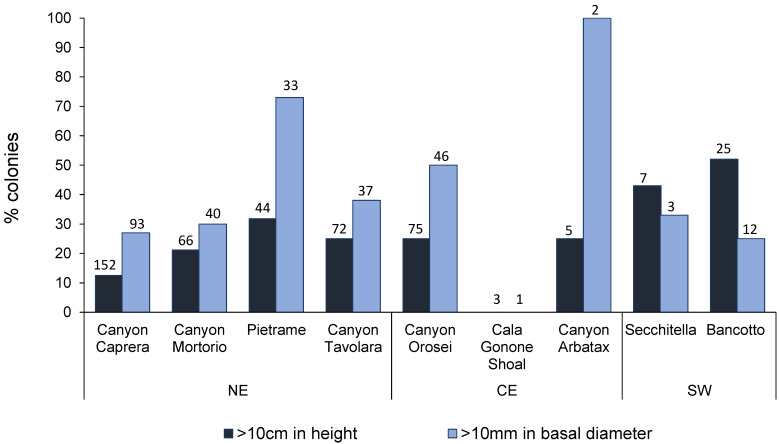
Percentage of red coral colonies taller than 10 cm (blue) and with basal diameter >10 mm (light blue). Numbers above the bars indicate the sample size. In Cala Gonone Shoal no colonies were >10 cm in height nor >10 mm in basal diameter.

Colony height was significantly higher in the SW area (12.6 ± 3.4 cm) than in the CE (7.8 ± 2.5 cm) and NE (6.3 ± 2.3 cm) areas (Table 3; PERMANOVA, *p* < 0.05). The maximum colony height (59 cm) was recorded in the SW area (Bancotto), while the minimum (0.8 cm) was recorded in the NE area (Tavolara Canyon). Overall, colony height did not show significant differences across the bathymetric range investigated (Figure 2B; K-W; *p* = ns); however, the Mann–Whitney pairwise comparison emphasized significant differences between the shallower colonies and the deepest ones (Supplementary Table S2). In addition, in the SW and CE areas, 50% and 27% of the colonies, respectively, were taller than 10 cm in height, whereas, at NE, only 20% of the population exceeded this size (Figure 3).

The average basal diameter ranged from 8.4 ± 0.3 mm in the SW area to 13.5 ± 8.8 in the CE area (Table 2). The maximum basal diameter (39.7 mm) was found in the CE area (Orosei Canyon), whereas the lowest one (2.1 mm) was found in the NE area (Caprera Canyon). The colony diameter did not change significantly with water depth (K-W; *p* = ns; Supplementary Table S3). The percentage of colonies with diameters larger than 10 mm, which is the minimum legal harvestable size in Sardinia (General Fisheries Commission for the Mediterranean, GFCM, 2011) was 51% in the CE area, 37% in the NE area, and 27% in the SW area (Figure 3).

**Figure 4:**
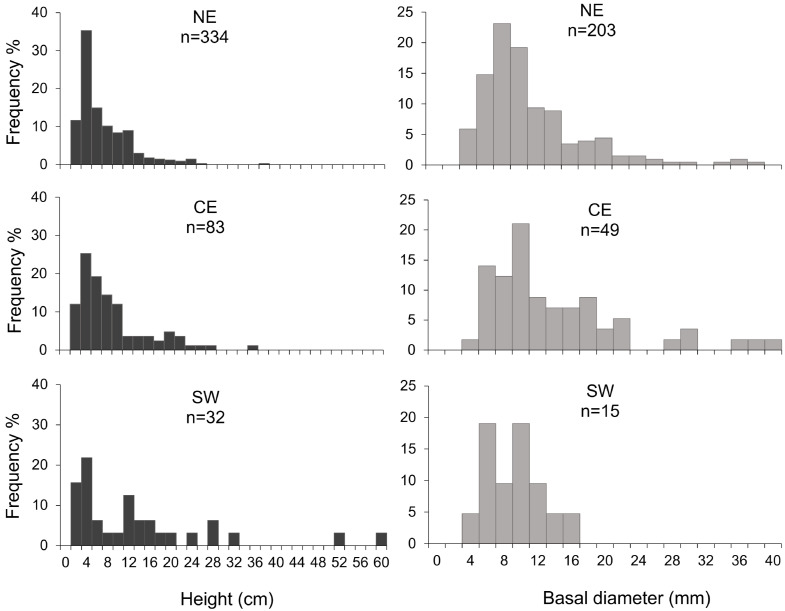
Size-frequency distribution of colony height (left column) and basal diameter (right column) of *C. rubrum* colonies in the three study areas.

In all the investigated areas, both height and basal diameter showed asymmetrical, non-normal distributions, with a higher percentage of colonies in the smaller-size classes (<4 cm and <8 mm for height and basal diameter, respectively; Figure 4). For both height and basal diameter, the frequency distributions appeared scattered, with some missing size classes. In all the areas, the size-frequency distribution of the height was unimodal, with the mode in the size class of 4 cm. The NE area displayed significant positive kurtosis (leptokurtic), indicating that distribution was slightly more peaked or overcentralized than the normal distribution. For the basal diameter, in the NE and CE areas, the distribution was unimodal, with a modal class of 8 and 10 mm, respectively, whereas in the SW zone, the distribution was polymodal (6 and 10 mm). In the NE area, the diameter distribution showed significant positive kurtosis and skewness, emphasizing a higher frequency of smaller-sized colonies.

In all the investigated areas, most of the colonies showed a simple branching pattern: in the NE and SW areas, 51% and 43% of the colonies, respectively, had first-order branches, whereas, in the CE area, 56% had second-order branches. Overall, the fifth-order branches were reached by only 2% of the colonies, with the highest percentage observed in the SW area (6%), and only when co-occurring with *E. cavolini*. There was a significant difference in the maximum level of branching among colonies dwelling in different depth categories (K-W; *p* = <0.001; ; Supplementary Table S4; Figure S2), with a higher proportion of colonies reaching the third-, fourth-, and fifth-order branches above 130 m depth rather than below.

Colony orientation did not significantly change between the sampling areas: ~50% of the colonies were oriented at 135° with respect to the hard bottom surface, while only 30% were perpendicular to the walls (oriented 0°; Table 2). The orientation of colonies (Figure S3) did significantly differ among the depth categories considered (K-W; *p* = <0.001; Supplementary Table S5).

**Figure 5:**
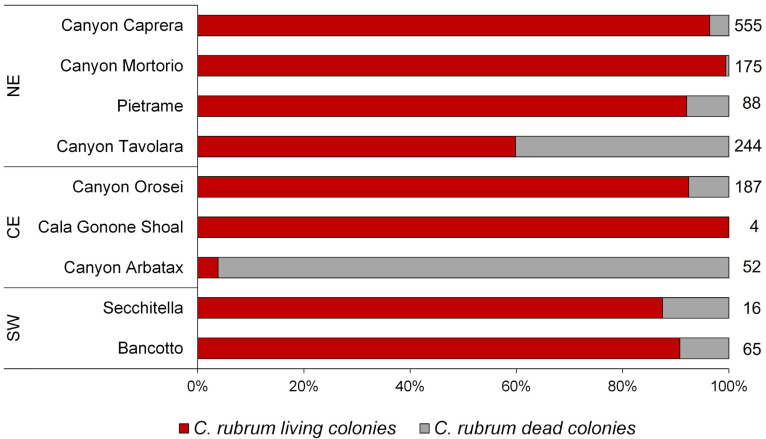
Percentage of living (red) and dead (grey) red coral colonies in each sampling site. Numbers next to the bars indicate the sample size.

Overall, an average of 80% of red coral colonies were alive at the moment of footage acquisition, with the highest percentage of living colonies (99%) observed in the site Pietrame and the lowest (4%) in the Arbatax Canyon, where, consequently, the highest percentage of dead colonies was registered (96%; Figure 5).

The frequency of frames in which fishing gears and/or nets were present was 1.3% in the NE area, 12.5% in the CE area, and 1% in the SW area. The highest fishing impact was recorded in the Cala Gonone Shoal and Arbatax Canyon, where lost fishing gears or nets were present in 16% and 18% of the frames, respectively. Overall, a negative correlation between the presence of fishing gear and the presence of red coral was found (Figure S4).

### 3.2. Eunicella cavolini: Occupancy, Density, and Population Size Structure

Overall, a total of 1481 yellow gorgonian colonies were found in 292 frames out of the 835 analyzed (35%) between 76 and 199 m depth. The average density ranged from 2.6 ± 3.7 to 3.3 ± 4.9 colonies m^−2^ in the CE and NE areas, respectively (Table 4). The maximum colony density (32 colonies m^−2^) was recorded in the Tavolara Canyon at 143 m depth and the lowest one (0.03 colonies m^−2^) in the Orosei Canyon at 92 m depth. Colony density was significantly lower above 111 m depth than below (Figure 2C; K-W, *p* < 0.001; Supplementary Table S6).

Colony height was higher in the SW (25.9 ± 10.3 cm) and CE (24.8 ± 14.6 cm) areas and lower in the NE area (20.1 ± 9.4 cm; Table 4). The maximum (71 cm) and minimum (3.4 cm) colony heights were recorded in the Orosei Canyon at 107 and 173 m depth, respectively. Colony height did not significantly vary with depth (Figure 2D; K-W, *p* = ns) despite the significant differences found through the pairwise comparison between shallower colonies (90–110) and those dwelling at greater depths (Supplementary Table S7).

The height-frequency distribution of *E. cavolini* populations appeared to be positively skewed, indicating the prevalence of the smaller height classes in the populations of all sampling areas (Figure S5). Populations from the NE and CE areas displayed a positive kurtosis value, suggesting a dominance of one of the smaller height classes (10–20 cm). The number of colonies with a height >30 cm was generally low (on average 23%), with the highest value observed in the CE area (32%), even if an important proportion of large colonies (20–30 and 30–40 cm) were found in most of the sites.

Colony orientation changed among areas: in the NE and CE zones, most colonies were oriented 90° with respect to the rocky walls (44% and 63% respectively), whereas in the SW area, 37% of them were 0°, and 37% were oriented 135° (Table 4). Furthermore, orientation varied significantly among the depth categories considered (K-W; *p* < 0.001; Supplementary Table S8).

### 3.3. Co-Occurrence of C. rubrum with E. cavolini

Overall, the co-occurrence of *C. rubrum* and *E. cavolini* was observed in 105 frames out of the total 835 analyzed frames (13%). Co-occurrence was observed throughout a wide bathymetric range (i.e., 91–199 m depth) but mostly in the deepest sites (120–160 m depth), where > 80% of frames showed the co-occurrence of the two species. We here found that 42% of the frames where red coral was present, also showed the presence of the yellow gorgonian, with the highest co-occurrence rate (93%) found in the Pietrame Canyon.

**Figure 6:**
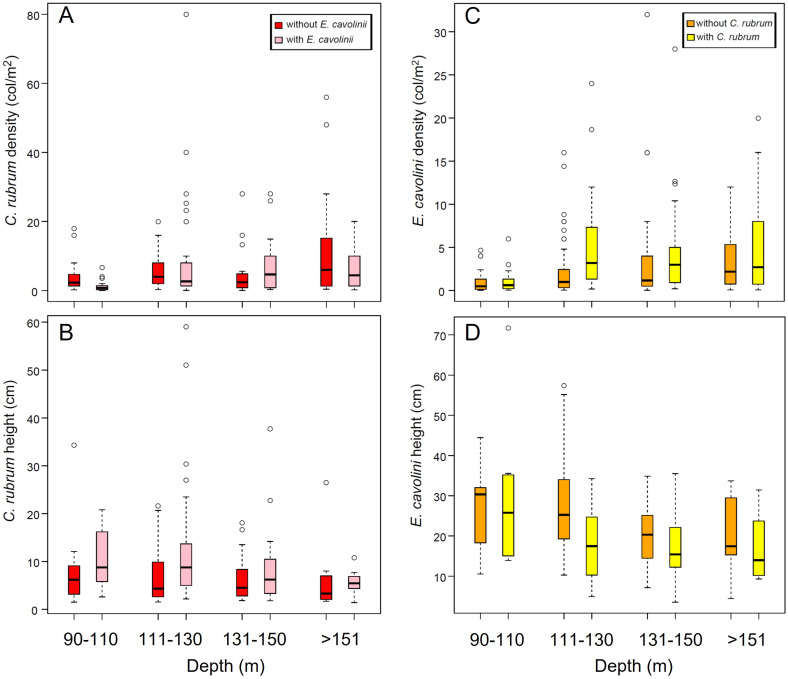
Boxplots showing colony density and height of (**A**,**B**) *C. rubrum* and (**C**,**D**) *E. cavolini* with and without the presence of the other species, at different depth ranges. The small circles are the outliers.

The density and height of the two species varied along the bathymetric range investigated, both in the presence and absence of the other species (Figure 6). Overall, both red coral density and height were higher in presence of *E. cavolini* (Figure 6A,B), with significantly higher values of height when yellow gorgonian was present (Table 3; PERMANOVA, *p* < 0.001). The basal diameter of red coral showed similar values with or without *E. cavolini*, except for the Mortorio Canyon, where it was significantly lower in the presence of the yellow gorgonian (Table 3; PERMANOVA, *p* < 0.05). In addition, the maximum branching level significantly changed with the presence of *E. cavolini* (Table 3; PERMANOVA, *p* < 0.001): red coral presented a higher proportion of colonies with the second-order branches (39% vs. 29%) and with the fifth-order branches when it co-occurred with *E. cavolini*.

For *E. cavolini*, density was significantly higher (Figure 6C; Table 3; PERMANOVA, *p* < 0.01), whereas height was lower when it co-occurred with the red coral (Figure 6D).

The results of the DistLM analysis, performed separately on both *E. cavolini* and *C. rubrum* density data, revealed that when the two species co-occurred, the density of one species was significantly correlated with the density of the other one (*p* < 0.001; explained variance: ~20–26%), and to a much less extent with water depth (*p* < 0.05: ~3%; Table 5). The effect of colony height on density was not significant (Table 5; Supplementary Table S9).

## 4. Discussion

### 4.1. Distribution and Population Structure of Deep-Dwelling C. rubrum

This study provides new data regarding the distributions of deep-dwelling *C. rubrum* and *E. cavolini* in Sardinia (Figure S6), which is also one of the most important and productive areas of red coral in the Western Mediterranean Sea. *C. rubrum* typically shows a patchy distribution, and the frequency of patches and density within patches can vary among investigated areas. In our study, density was noticeably lower than in other deep-sea areas [17], except for some sites located in the Southern Tyrrhenian Sea (Gulf of Naples), where similarly low values were recorded [34]. Differences in density and population structure in the deep sea are mainly related to different abiotic factors, such as the low temperature, which can affect the biology of red coral, slowing down the development of male and female gonads and increasing the annual prey capture rates (see reference [14] and references therein). Moreover, red coral population structure can also be driven by “self-thinning” processes: an intra-specific competition process by which space limitations link population density to the mean individual ground cover [53,54]. Thus, immature or disturbed populations should be characterized by high densities, while mature or stable populations should be composed of big and sparse colonies [34,54]. In light of the uncontrolled and destructive harvesting practices that took place over the last two centuries, high-density populations are usually observed in shallow water, whereas low-density and mature populations are found in deeper ones [14]. However, our results indicate an opposite pattern, since we observed higher densities and lower sizes towards deeper sites. This pattern could be explained by considering that besides human impacts, different environmental factors and/or disturbances may drive the population structure. In this perspective, most of our sampling sites were located along submarine canyons, where the frequency of disturbance due to, e.g., dense water cascading and turbidity flow, can be more incisive. These events can negatively affect the stability of larger/older colonies and lead to a shift towards higher densities and smaller sizes, as already documented in sites showing similar ecological features in Sardinia [54]

**Figure 7:**
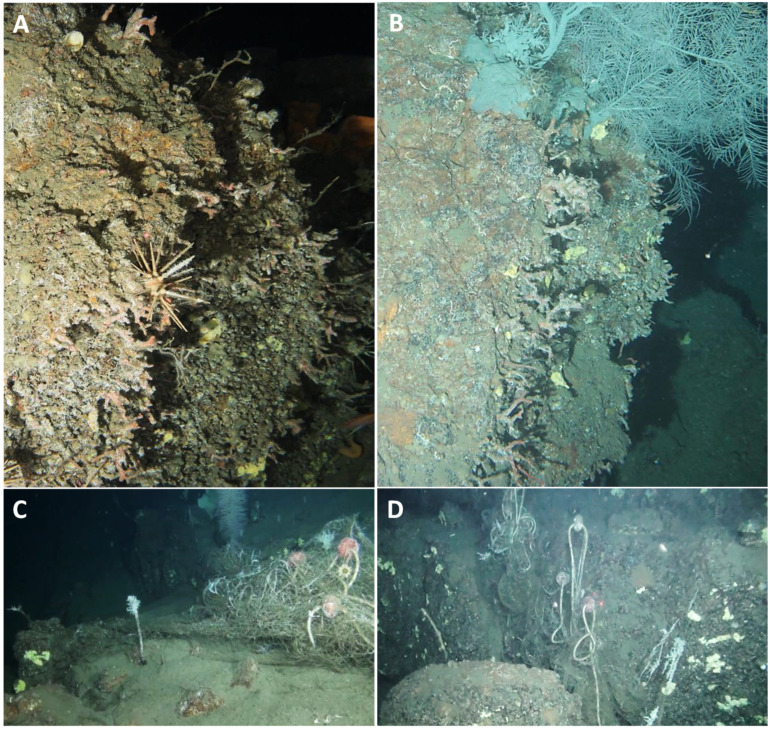
Dead colonies of *Corallium rubrum* (**A**,**B**) and lost fishing gears (**C**,**D**) found along the Arbatax Canyon (CE area).

The demographic analysis revealed that most of the surveyed red coral populations were characterized by height frequency distribution skewed towards smaller-sized colonies, mostly 4 cm in height, which is smaller than the size at which colonies are supposed to reach 100% fertility [42,55,56]. It is noteworthy that some size classes were missing (see Figure 4), which could be an indication of past disturbances [57,58,59]. This hypothesis is also corroborated by the high percentage of dead colonies found along the Arbatax Canyon, where entire patches of dead colonies showed evident branch breakages (Figure 7A,B). Along this canyon, we indeed found a high % of frames (18%) showing the presence of lost fishing gears that document an intense fishing activity within the area (Figure 7C,D). This kind of anthropogenic pressure is very common in the Tyrrhenian Sea [34,60] and could negatively affect benthic communities both directly and indirectly: (i) mechanical friction can directly impact arborescent colonies, eradicating larger and taller ones, provoking branch breakages, and inducing diseases; (ii) generated resuspension may lead to an increase of daily sediment fluxes and sedimentation rates along the canyon, which can negatively affect the settlement, recruitment, and survival of red coral colonies [61,62,63]. Although the present dataset has no evidence of this phenomenon, it is worth noting how such stressors may in turn also trigger infections by pathogens to more sensitive colonies [34].

Overall, the red coral population here investigated showed a basal diameter frequency distribution positively skewed, and an averaged basal diameter of 10 mm, indicating that most of the colonies are young and in the growth phase [19,57]. This is also confirmed by the analysis of the branching pattern showing a low morphological complexity of red coral colonies: most of them, indeed, presented only the first-order branches, which are known to be produced at an average age of about 10 years [44]. On the contrary, the fifth-order branches were observed only in 2% of the total measured colonies.

Overall, 39% of colonies exceeded the minimum size for harvesting (10 mm in basal diameter, as imposed by Sardinian management measures, stricter than those recommended within the GFCM competence area). This is a lower value compared to that found (46%) in a previous paper focused on deep-dwelling red coral colonies in the Tyrrhenian Sea [17]; however, in that paper, the authors referred to the minimum legally harvestable size of basal diameter (7 mm) imposed by the GFCM. The presence of larger colonies is important for population survival, as the reproductive output increases with the size [55,56]. Our data suggest that the stricter management measures imposed by Sardinia since 1979 [64] have guaranteed an overall good conservation status of the resource, but more effective tools, such as the use of onboard scientific observers, should be considered and implemented in the future [23]. In addition, considering that deep-sea banks are now the main target of professional harvesting and that deep red coral populations are genetically different and separated from shallow-water ones by an apparent barrier to gene flow [16,24], it becomes urgent to also develop new management measures to specifically protect deep-sea coral populations.

### 4.2. Demography of Deep-Dwelling E. cavolini

Our results contribute to advancing knowledge on the distribution and demographic features of populations of *E. cavolini* dwelling up to 200 m depth. Overall, density values of the yellow gorgonian were lower than those previously found in shallow-water ecosystems [28,65] and mesophotic habitats [66]. However, similar low-density values were reported from other deep-sea sites located at 120 m depth in the Tyrrhenian Sea [29]. We also document an inverse relation between colony density and maximum height (Figure 2C,D), supporting the hypothesis that recruitment can be driven by intra-specific competition mechanisms and can thus increase when large colonies are absent [28]. In addition, we also found that density increased with depth, while height decreased. Like many other octocorals, *E. cavolini* shows high phenotypic plasticity, modulating the size, shape, and orientation of the colonies in response to water movement [67,68,69]. Thus, the increasing presence of crowded, small colonies (<20 cm) found with the increasing of water depth may be caused by local environmental conditions, such as the higher frequency of disturbance (in terms of dense water cascading and water flow), which is known to characterize deep-sea canyons [70].

### 4.3. Patterns of Co-Occurrence between Red Coral and Yellow Gorgonian in the Deep Mediterranean Sea

In this study, we found a recurrent association (42% of frames where red coral was present) between red coral and the yellow gorgonian, especially below 120 m depth. These results indicate the two species can partially overlap their distribution in deep sublittoral areas, thus potentially competing for space and resources. Although red coral is known to form monospecific patches, its association with other gorgonians in the deep Mediterranean Sea is not completely unexpected; indeed, in the Tyrrhenian Sea, red coral has been previously found associated with *E. cavolini* and *Paramuricea clavata* (60% of ROV frames) [17]. Furthermore, in the Northeast of Spain, in the Medes Islands Marine Protected Area, *C. rubrum* and the scleractinian cup coral, *Leptopsammia pruvoti,* usually tend to segregate one from the other at a small spatial scale, but in some cases, the latter wedged within crowded *C. rubrum* patches, probably due to the aggressive behavior of *Leptopsammia pruvoti* [8].

We report here that when the two species co-occurred, the density of both red coral and yellow gorgonian were higher and significantly influenced by the density values of the other, while colony size (i.e., the average height of colonies) or depth were less or not significant factors. Our results suggest that competition for space and settlement, likely driven by very similar larval preferences, could be the major constraint for the co-existence of these two suspension feeders. However, the higher density values found when the two species co-occurred, could also open new questions regarding the potential positive effects as foundation species, that are able to modify the peculiar habitat where they live (i.e., reducing water flow velocity, increasing the quantity and quality of organic matter). In addition, we also observed a decrease in the height of *E. cavolini*, with increasing density when occurring with red coral: this pattern could be likely ascribed to intra-specific self-thinning processes driven by increased density values but also to inter-specific relations, for which we acknowledge the limitation of the present dataset, which misses a temporal replication of observations that would ultimately contribute to understanding such a morphological evolution of colonies. In conclusion, the observed association could be linked to abiotic variables presumably joined with biotic ones [28,71]: different factors not controlled in this study, amongst which similar larval preferences for peculiar features of the rocky bottom, along with inter-specific interactions, may drive the distribution of both species at small spatial scale.

## 5. Conclusions

The present study advances our knowledge on the distribution and population structures of deep-dwelling *C. rubrum* and *E. cavolini* populations (Figure S6). Our data confirm the presence of an inverse size–density relation for both species, with the highest densities found at greater depths. Most of the colonies were alive at the moment of sampling, with the exception of those dwelling along the Arbatax Canyon, which appeared to have suffered from a massive mortality event. Considering that red coral populations are deeply impacted by direct and indirect anthropogenic activities (i.e., fishing, contamination, and harvesting) and that deep-sea banks are now the main target of professional fishermen, there is an urgent need to develop new and effective management measures to guarantee the protection and conservation of such populations and their associated biodiversity.

This paper also reports the co-occurrence of red coral and yellow gorgonian between 90 and 200 m depth, demonstrating a partially overlapping distribution and that the variability of the two species’ densities is significantly driven by the density of the other. However, further studies are needed to better clarify the relative effects on crucial biological features, such as settlement and recruitment, and to analyze the role of different environmental factors potentially influencing these inter-specific relationships.

## Figures and Tables

**Table 1:** Details of the investigated sites during ROV survey: geographic area, sites’ names, dive id number, depth range, coordinates, surveyed area, and total number of frames from each dive.

Geographic Area	Site	Dive ID Number	Depth Range (m)	Coordinates		Surveyed Area (m^2^)	N Frames
				Lat (N)	Long (E)		
North-east	Canyon Caprera	9	112–220	41°17′427″	9°37′481″	52	10
	Canyon Caprera	10	127–190	41°18′357″	9°38′021″	84	28
	Canyon Caprera	14	121–165	41°20′433″	9°38′121″	1068	79
	Canyon Mortorio	11	51–123	41°04′217″	9°47′899″	107	87
	Canyon Mortorio	12	110–152	41°04′152″	9°47′845″	140	114
	Canyon Mortorio	13	126–163	41°04′200″	9°48′224″	122	48
	Pietrame	7	144–150	40°43′080″	9°49′933″	86	35
	Canyon Tavolara	8	106–190	40°55′169″	9°54′140″	456	69
	Canyon Tavolara	15	90–103	40°54′858″	9°53′592″	97	20
	Canyon Tavolara	16	107–170	40°54′860″	9°54′041″	225	63
	Canyon Tavolara	17	158–290	40°54′768″	9°54′908″	264	70
Central-east	Canyon Orosei	2	76–120	40°21′825″	9°53′670″	661	89
	Canyon Orosei	4	96–120	40°21′767″	9°53′657″	18	5
	Canyon Orosei	5	156–187	40°21′692″	9°53′950″	171	57
	Cala Gonone Shoal	19	54–217	40°17′544″	9°40′252″	71	32
	Canyon Arbatax	20	108–147	39°58′136″	9°43′734″	73	14
	Canyon Arbatax	21	121–181	39°58′080″	9°43′770″	213	43
South-west	Secchitella	33	143–156	39°10′122″	8°06′133″	78	25
	Bancotto	29	108–140	38°44′425″	8°29′025″	208	49

**Table 2:** Summary of data on red coral *Corallium rubrum* in the investigated sites: area, site, and depth range where the colonies were found, average colony densities, mean height, mean basal diameter, maximum order of branches, the orientation of colonies, and the portion of colonies occurring with the yellow gorgonian. * = one measured value (one frame with only one measurable colony for basal diameter); na = not available; information not possible to acquire.

Area	Site	Depth Range (m)	Colony Density (col/m^2^)	Height (cm)	Basal Diameter (mm)	Max Order Branches	Orientation of Colonies (%)					Colonies Occurring with *E. cavolini* (%)
							0°	45°	90°	135°	180°	
North-East	Canyon Caprera	116–152	2.1 ± 1.9	5.3 ± 4.4	0.84 ± 0.41	fifth	0	0	30	56	14	85
	Canyon Mortorio	108–146	6.9 ± 8.7	6.14.9	0.95 ± 0.37	fourth	6	6	55	31	2	19
	Pietrame	145–149	3.2 ± 0.4	9.4 ± 6.8	1.70 ± 0.90	fourth	0	0	21	48	31	93
	Canyon Tavolara	108–257	3.7 ± 3.6	6.5 ± 4.6	1.04 ± 0.56	fourth	0	1	30	62	7	29
Central-East	Canyon Orosei	91–176	6.2 ± 10.8	8.5 ± 6.9	1.34 ± 0.90	third	0	1	13	63	23	37
	Cala Gonone Shoal	132	4 *	3.5 ± 0.8	0.75 *	third	0	0	0	100	0	100
	Canyon Arbatax	131–148	8.1 ± 7.5	10.8 ± 4	1.91 ± 0.24	na	0	4	50	44	2	0
South-west	Secchitella	148–152	1.6 ± 2.1	7.9 ± 9.1	0.76 ± 0.56	third	0	11	22	56	11	19
	Bancotto	112–123	3.5 ± 1.6	13.9 ± 14.9	0.86 ± 0.28	fifth	9	6	42	26	17	34

**Table 3:** Results of the PERMANOVA analysis testing for differences in: (A) red coral density, height, basal diameter, maximum branching level, and colony orientation, and in (B) yellow gorgonian density, height, and colony orientation, among areas (Ar), between sites (Si)within areas and co-occurrence or not (Pr) within sites for each area. df = degrees of freedom; MS = mean square; Pseudo-F = permutational F; *** = *p* < 0.001; ** = *p* < 0.01; * = *p* < 0.05; ns = not significant.

**(A) Variable**	**Source**	**df**	**MS**	**Pseudo-F**	***p* (MC)**
**Density**	Ar	2	69.010	1.022	ns
	Si(Ar)	6	53.380	0.443	ns
	Pr(Si(Ar))	7	138.100	1.799	ns
	Residual	233	76.720		
	Total	248			
**Height**	Ar	2	174.170	3.362	*
	Si(Ar)	6	56.693	0.351	ns
	Pr(Si(Ar))	7	233.400	6.345	***
	Residual	433	36.785		
	Total	448			
**Basal diameter**	Ar	2	0.430	1.0020	ns
	Si(Ar)	6	0.542	0.859	ns
	Pr(Si(Ar))	7	0.786	2.162	*
	Residual	251	0.364		
	Total	266			
**Maximum branching level**	Ar	2	3.027	1.627	ns
	Si(Ar)	6	5.701	1.795	ns
	Pr(Si(Ar))	7	3.988	5.186	***
	Residual	1352	0.769		
	Total	1367			
**Colony orientation**	Ar	2	1899.5	0.480	ns
	Si(Ar)	6	14543	1.551	ns
	Pr(Si(Ar))	7	12243	13.546	***
	Residual	1352	903.82		
	Total	1367			
**(B) Variable**	**Source**	**df**	**MS**	**Pseudo-F**	***p* (MC)**
**Density**	Ar	2	3.295	0.145	ns
	Si(Ar)	7	30.741	0.967	ns
	Pr(Si(Ar))	8	42.990	2.412	**
	Residual	274	17.822		
	Total	291			
**Height**	Ar	2	101.430	0.812	ns
	Si(Ar)	7	130.450	1.043	ns
	Pr(Si(Ar))	8	128.550	1.052	ns
	Residual	246	122.250		
	Total	263			
**Colony orientation**	Ar	2	4606.100	0.360	ns
	Si(Ar)	7	44923.000	7.227	***
	Pr(Si(Ar))	8	16681.000	9.614	***
	Residual	1426	1735.100		
	Total	1443			

**Table 4:** Summary of data on the yellow gorgonian *Eunicella cavolini* in the investigated sites: area, site, depth range where the colonies were found, average colony densities, mean height, orientation of colonies, and portion of colonies occurring with the red coral.

Area	Site	Depth Range (m)	Colony Density (col/m^2^)	Height (cm)	Orientation of Colonies (%)					Colonies Occurring with *C. rubrum* (%)
					0°	45°	90°	135°	180°	
North-East	Canyon Caprera	122–152	4.5 ± 5.6	19.1 ± 10.4	30	18	32	20	0	55
	Canyon Mortorio	109–151	1.9 ± 1.4	24.9 ± 12.3	3	22	49	27	0	18
	Pietrame	142–149	2.1 ± 2.3	21.2 ± 7.7	0	17	41	41	2	67
	Canyon Tavolara	109–199	2.5 ± 6	18.5 ± 9.5	22	15	54	9	0	40
Central-East	Canyon Orosei	76–187	2.5 ± 3.6	24.1 ± 13.6	5	7	48	39	1	14
	Cala Gonone Shoal	114–175	3.6 ± 4.6	26.9 ± 12.5	0	11	78	11	0	7
South-west	Secchitella	149–152	3.5 ± 4.3	10.9 ± 4.8	0	0	5	74	21	42
	Bancotto	108–126	2.6 ± 2.8	24.8 ± 11.4	74	23	3	0	0	28

**Table 5:** Results from the sequential test of the distance-based multivariate analysis for a linear model (DistLM). The following abbreviations are used: *** = *p* < 0.001; * = *p* < 0.05; ns = not significant; Prop. (%) = percentage of explained variation; Cumul. (%) = cumulative percentage of total variation.

Variables	R^2^	*p*-Value	Prop. (%)	Cumul. (%)
**(A) *C. rubrum* density**				
**Density of *E. cavolini***	0.267	***	26.7	26.7
**Depth**	0.299	*	3.2	29.9
**Height of *E. cavolini***	0.299	ns	0.04	29.9
**(B) *E. cavolini* density**				
**Density of *C. rubrum***	0.194	***	19.4	19.4
**Depth**	0.227	*	3.3	22.7
**Height of *C. rubrum***	0.231	ns	0.4	23.1

## Data Availability

Data are contained within the article or supplementary material.

## Supplementary Materials

The following supporting information can be downloaded at: www.mdpi.com/article/10.3390/biology11020333/s1, Figure S1: Example of analyzed frame; Figure S2: Pie chart representing the relative percentages of the highest level of branching in *C. rubrum* colonies, across the depth-categories investigated; Figure S3: Pie chart representing the orientation of *C. rubrum* colonies (expressed as relative %), across the depth-categories investigated; Figure S4: Relationship between the presence of red coral vs fishing impact; Figure S5: Size-frequency distribution of *E. cavolini* colony height in the three investigated areas; Figure S6: distribution map of the two investigated species based on the data obtained during the study. Table S1: Output of the Mann-Whitney pairwise comparison on differences in *C. rubrum* density among the different depth-categories; Table S2: Output of the Mann-Whitney pairwise comparison on differences in *C. rubrum* height among the different depth-categories; Table S3: Output of the Mann-Whitney pairwise comparison on differences in *C. rubrum* basal diameter among the different depth-categories; Table S4: Output of the Mann-Whitney pairwise comparison on differences in *C. rubrum* maximum branching pattern among the different depth-categories considered in this study; Table S5: Output of the Mann-Whitney pairwise comparison on differences in *C. rubrum* orientation among the different depth-categories; Table S6: Output of the Mann-Whitney pairwise comparison on differences in *E. cavolini* density among the different depth-categories; Table S7: Output of the Mann-Whitney pairwise comparison on differences in *E. cavolini* height among the different depth-categories; Table S8: Output of the Mann-Whitney pairwise comparison on differences in *E. cavolini* orientation among the different depth-categories; Table S9: Results from the sequential test of the Distance based multivariate analysis for a Linear Model (DistLM) based on AIC criterium.
