# Supplementary material for: Deep-Dwelling Populations of Mediterranean Corallium rubrum and Eunicella cavolini: Distribution, Demography, and Co-Occurrence
Source: Biology (Basel). 2022 Feb 20;11(2):333. doi: 10.3390/biology11020333 (PMC8869470; doi:10.3390/biology11020333)
Supplement: Supplementary file 1 [file biology-11-00333-s001.zip › biology-1559894-supplementary.pdf]

SUPPLEMENTARY MATERIAL

Deep-Dwelling Populations of Mediterranean *Corallium rubrum* and *Eunicella cavolini*: Distribution, Demography, and Co-occurrence

Laura Carugati [1,*], Davide Moccia [1], Lorenzo Bramanti [2], Rita Cannas [1], Maria Cristina Follesa [1], Susanna Salvadori [1] and Alessandro Cau [1]

[1] Department of Life and Environmental Sciences, University of Cagliari, Via T. Fiorelli 1, 09126 Cagliari, Italy; mocciadavide@unica.it (D.M.); rcannas@unica.it (R.C.); follesac@unica.it (M.C.F.); salvador@unica.it (S.S.); alessandrocau@unica.it (A.C.)

[2] Laboratoire d'Ecogeochimie des Environnements Benthiques (LECOB), Sorbonne Universites, Université Pierre-et-Marie-Curie, Centre National de la Recherche Scientifique (CNRS), Observatoire Oceanologique, F-66650 Banyuls sur Mer, France; lorenzo.bramanti@obs-banyuls.fr

* Correspondence: laura.carugati@unica.it

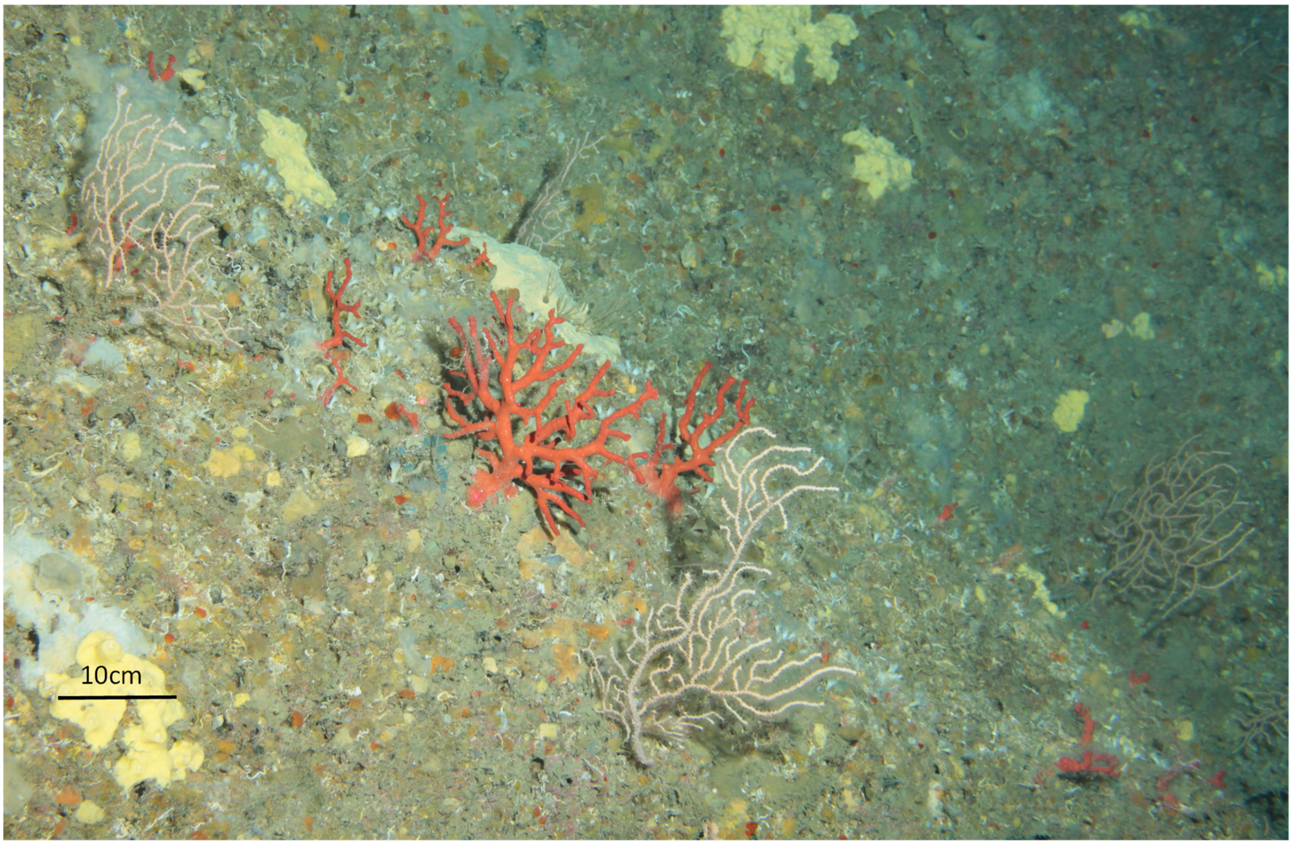

Figure S1. Example of frame used in this study with the reference scale provided by the red coloured laser beams.

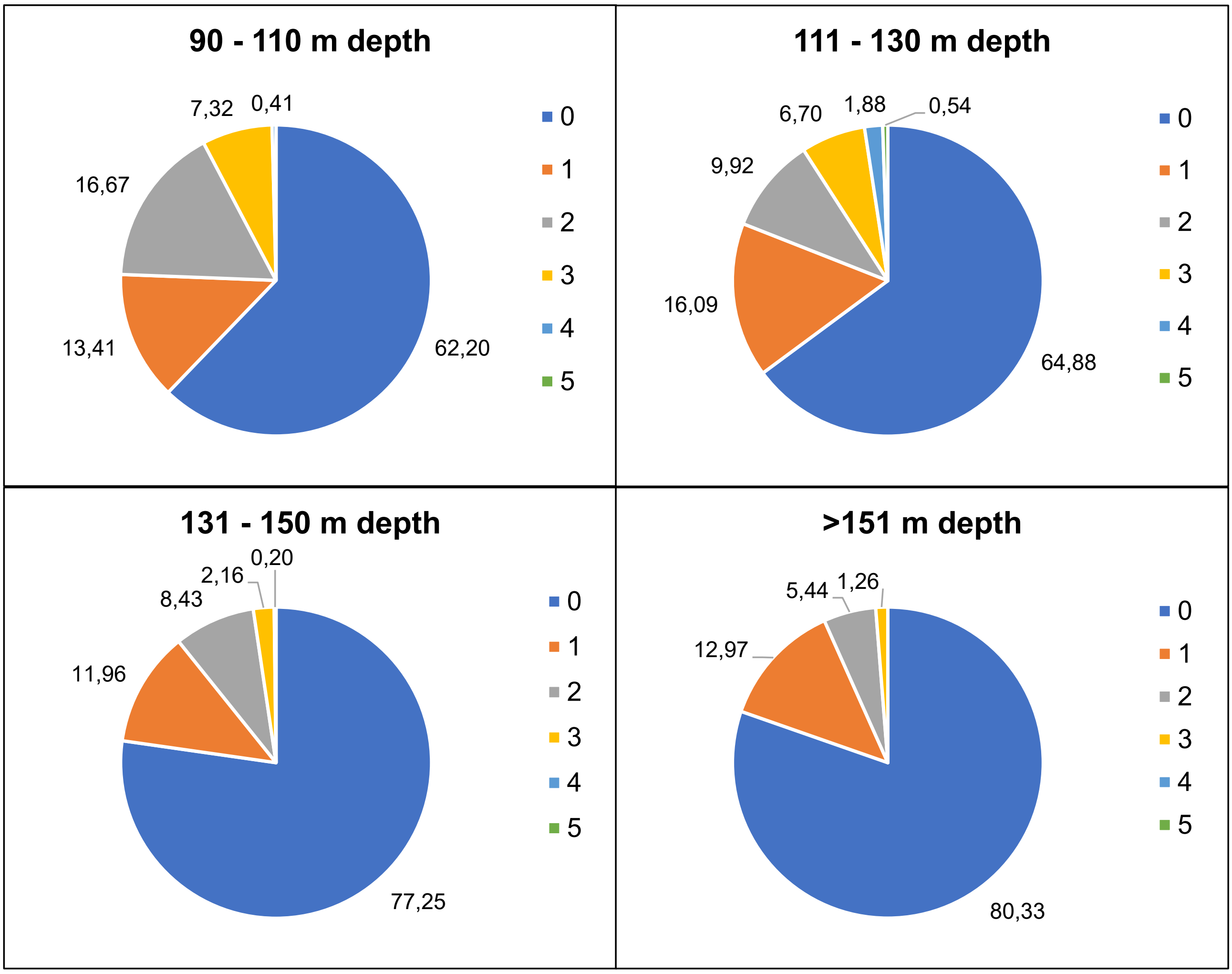

Figure S2. Pie chart representing the relative percentages of the highest level of branching in *C. rubrum* colonies, across the depth-categories investigated. The number in the legend corresponds to the maximum branching pattern.

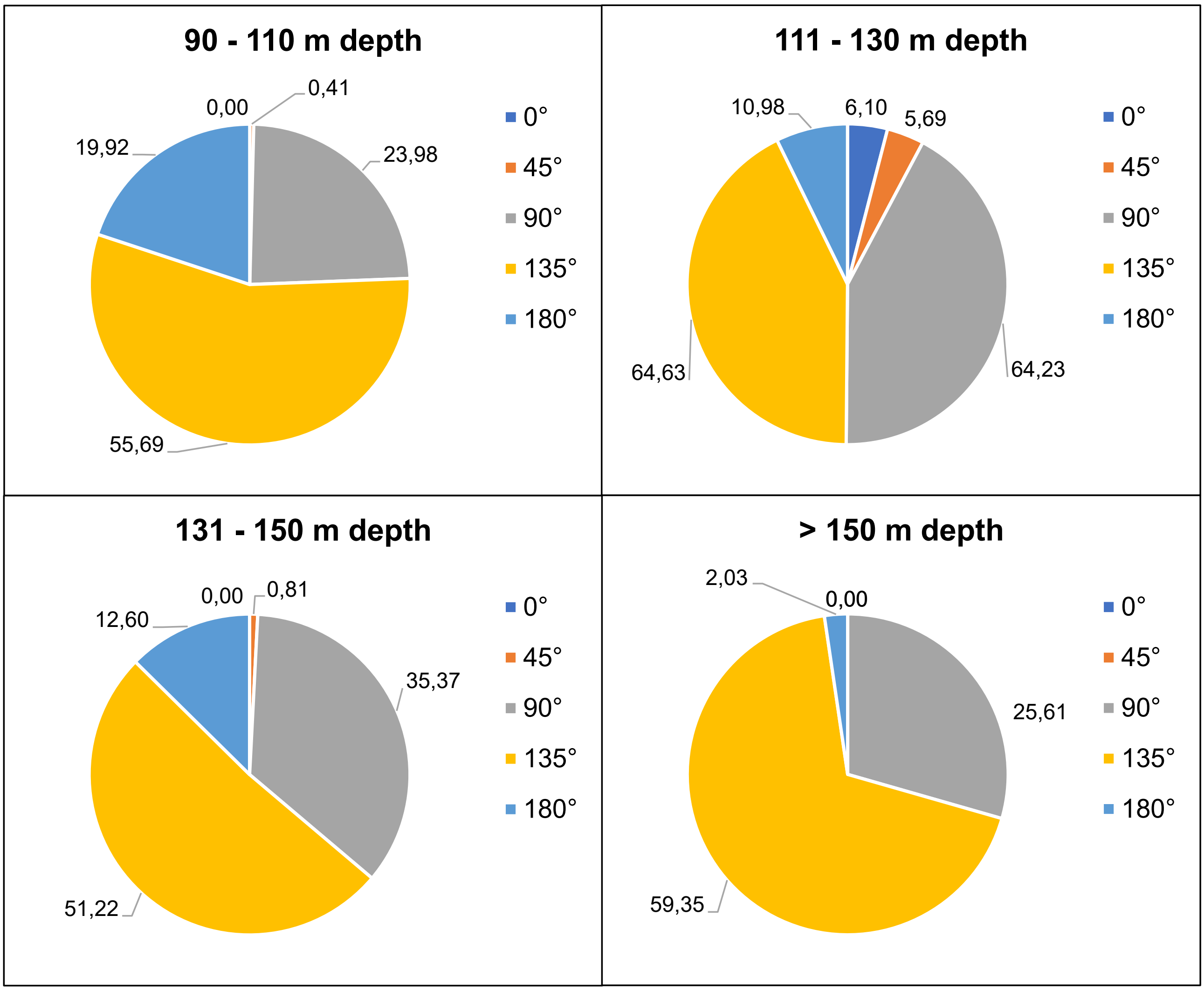

Figure S3. Pie chart representing the orientation of *C. rubrum* colonies (expressed as relative %), across the depth-categories investigated.

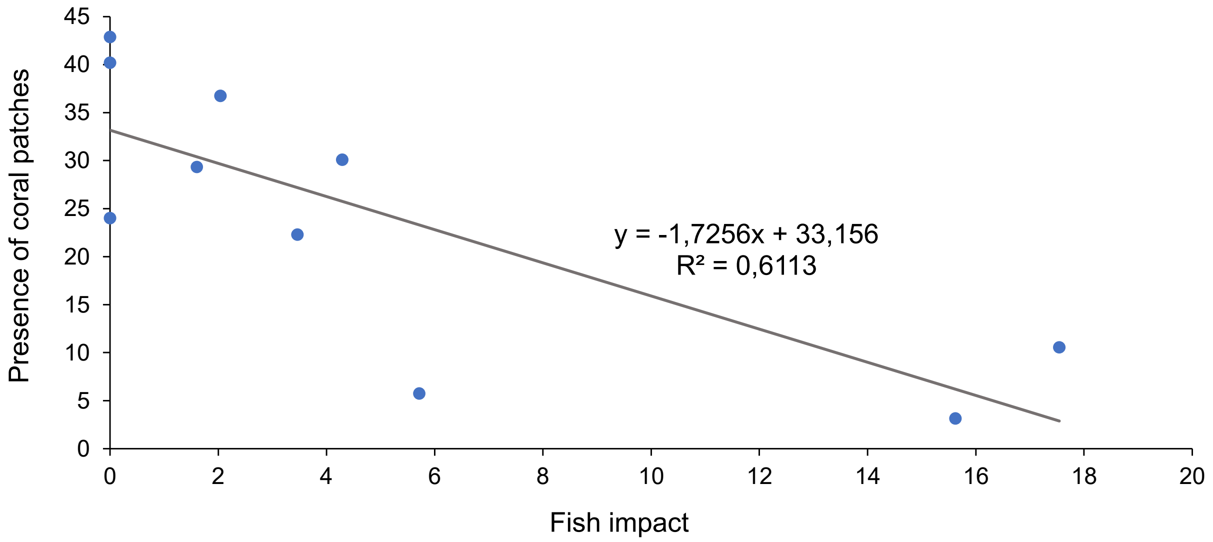

Figure S4. Relationship between the presence of red coral (expressed as % of analyzed frames including red coral colonies), vs fishing impact (expressed as % of analyzed frames including lost fishing gears) in the investigated areas (n=10, R=0.783, p<0.01).

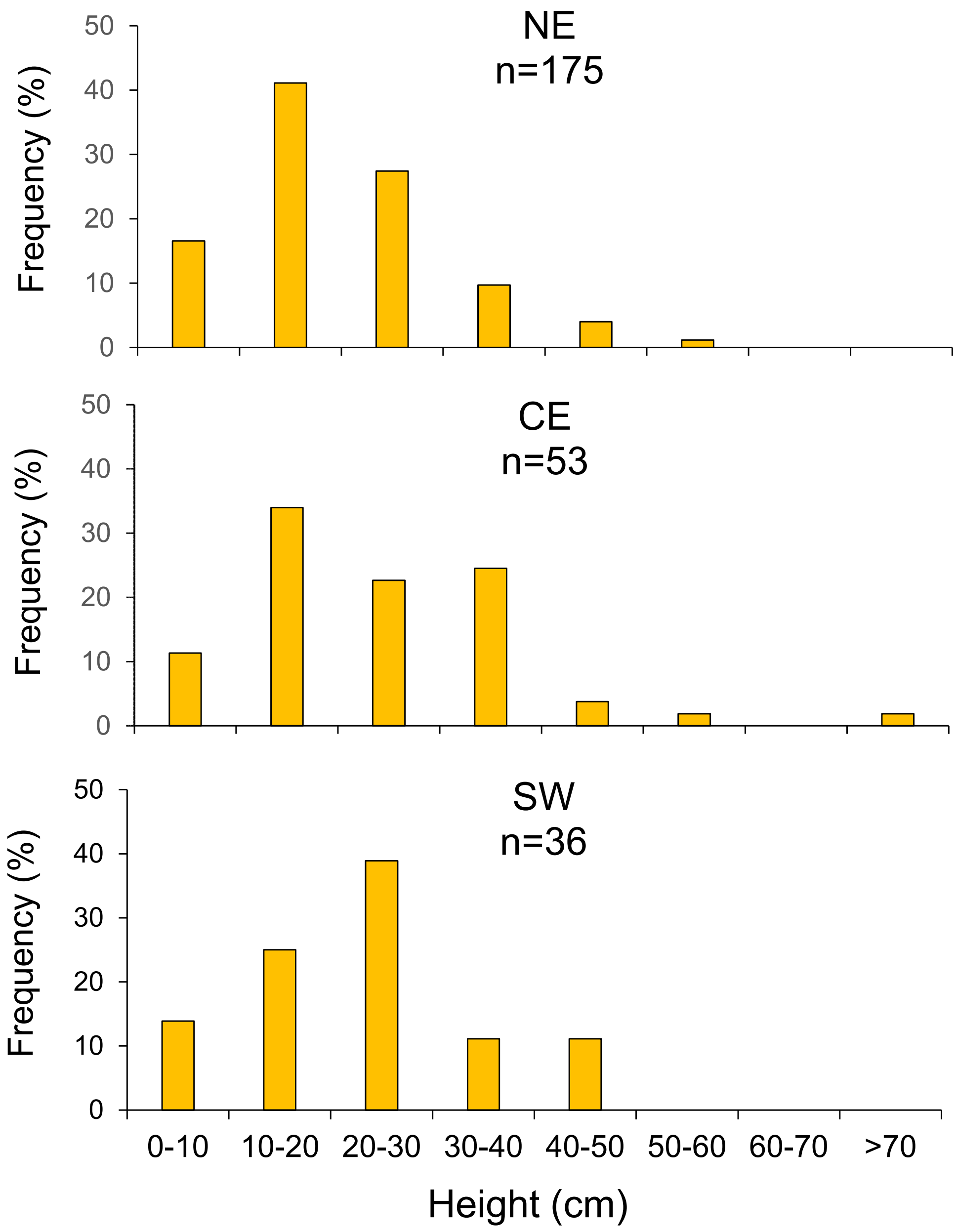

Figure S5. Size-frequency distribution of *E. cavolini* colony height in the three investigated areas.

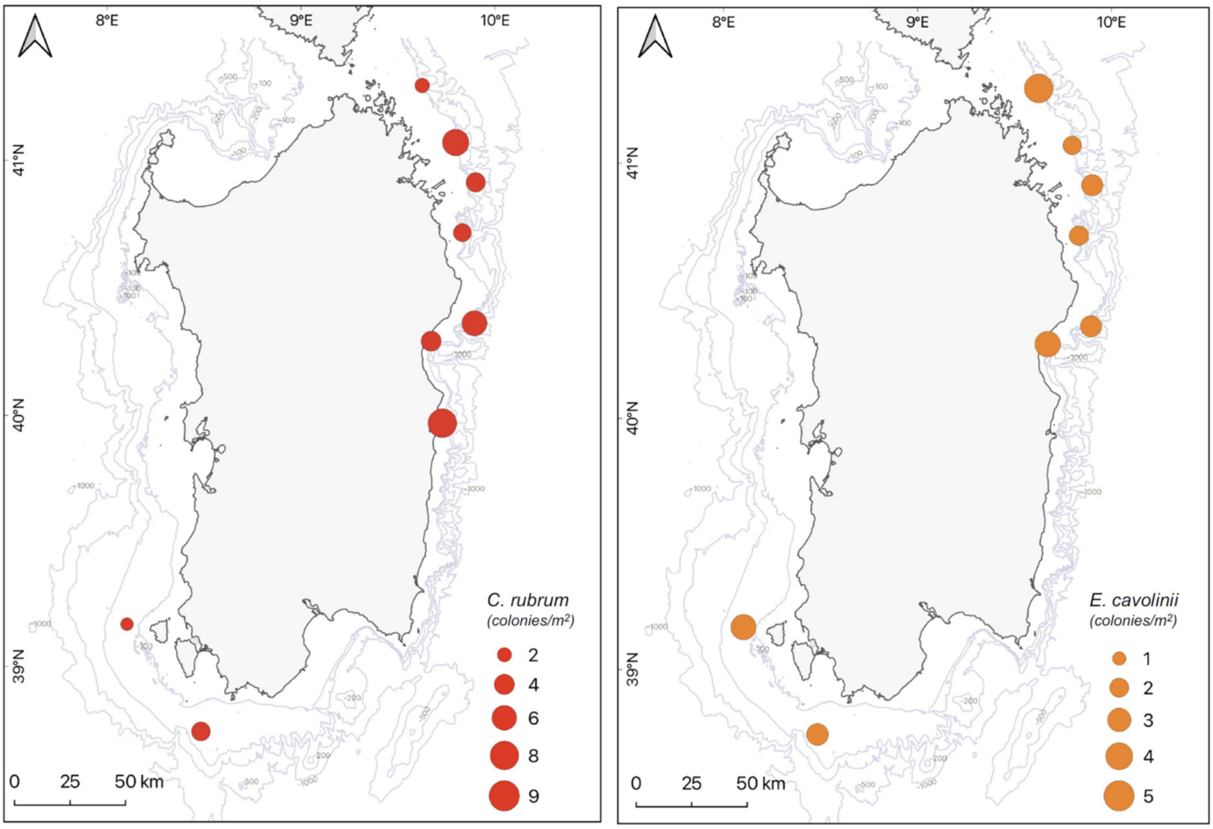

Figure S6. Distribution map of the two investigated species (*C. rubrum* on the left, *E. cavolini* on the right) based on the data obtained during the study.

Table S1. Output of the Mann-Whitney pairwise comparison on differences in *C. rubrum* density among the different depth-categories considered in this study.

	90-110	111-130	131-150	> 150
**90-110**		**0.00085**	**0.02872**	**0.00394**
**111-130**	**0.00085**		0.69350	0.28230
**131-150**	**0.02872**	0.69350		0.15930
**>151**	**0.00394**	0.28230	0.15930	

Table S2. Output of the Mann-Whitney pairwise comparison on differences in C. rubrum height among the different depth-categories considered in this study.

	90-110	111-130	131-150	> 150
**90-110**		0.92760	0.24580	**0.01325**
**111-130**	0.92760		0.37240	**0.02965**
**131-150**	0.24580	0.37240		0.11890
**>151**	**0.01325**	**0.02965**	0.11890	

Table S3. Output of the Mann-Whitney pairwise comparison on differences in *C. rubrum* basal diameter among the different depth-categories considered in this study.

	90-110	111-130	131-150	>151
**90-110**		**0.04399**	0.09740	**0.01951**
**111-130**	**0.04399**		0.88780	0.25860
**131-150**	0.09740	0.88780		0.25120
**>151**	**0.01951**	0.25860	0.25120	

Table S4. Output of the Mann-Whitney pairwise comparison on differences in *C. rubrum* maximum branching pattern among the different depth-categories considered in this study.

	90-110	111-130	131-150	>151
**90-110**		0.43890	**1.93E-06**	**8.46E-07**
**111-130**	0.43890		**0.00001**	**6.63E-06**
**131-150**	**1.93E-06**	**0.00001**		0.25650
**>151**	**8.46E-07**	**6.63E-06**	0.25650	

Table S5. Output of the Mann-Whitney pairwise comparison on differences in *C. rubrum* orientation among the different depth-categories considered in this study.

	**90-110**	**111-130**	**131-150**	**>151**
**90-110**		**2.64E-13**	**1.86E-01**	**2.27E-04**
**111-130**	**2.64E-13**		**1.73E-13**	**0.00002**
**131-150**	**1.86E-01**	**1.73E-13**		**0.00865**
**>151**	**2.27E-04**	**0.00002**	**0.00865**	

Table S6. Output of the Mann-Whitney pairwise comparison on differences in *E. cavolini* density among the different depth-categories considered in this study.

	**90-110**	**111-130**	**131-150**	**>151**
**90-110**		**3.60E-05**	**4.34E-06**	**2.45E-06**
**111-130**	**3.60E-05**		0.43480	0.16530
**131-150**	**4.34E-06**	0.43480		0.45890
**>151**	**2.45E-06**	0.16530	0.45890	

Table S7. Output of the Mann-Whitney pairwise comparison on differences in *E. cavolini* height among the different depth-categories considered in this study.

	**90-110**	**111-130**	**131-150**	**>151**
**90-110**		0.25130	**0.02636**	**0.04949**
**111-130**	0.25130		0.12060	0.24600
**131-150**	**0.02636**	0.12060		0.78110
**>151**	**0.04949**	0.24600	0.78110	

Table S8. Output of the Mann-Whitney pairwise comparison on differences in *E. cavolini* orientation among the different depth-categories considered in this study.

	**90-110**	**111-130**	**131-150**	**>151**
**90-110**		**0.00315**	**0.00007**	**2.03E-26**
**111-130**	**0.00315**		**2.01E-24**	**1.68E-68**
**131-150**	**0.00007**	**2.01E-24**		**8.03E-19**
**>151**	**2.03E-26**	**1.68E-68**	**8.03E-19**	

Table S9. Results from the sequential test of the Distance based multivariate analysis for a Linear Model (DistLM) based on AIC criterium. The following abbreviations are used: *** = P < 0.001; * = P < 0.05; ns = not significant; Prop. (%) percentage of explained variation; Cumul. (%) cumulative percentage of total variation.

**Variable**	**AIC**	**P-value**	**Prop. (%)**	**Cumul. (%)**
**A)** ***C. rubrum*** **density**				
**Density of** ***E. cavolini***	-176,95	***	26.7	26.7
**Height of** ***E. cavolini***	-175,07	ns	0.1	26.7
**Depth**	-177,71	*	3.2	29.9
**B)** ***E. cavolini*** **density**				
**Height of** ***C. rubrum***	-189,9	ns	3.5	3.5
**Density of** ***C. rubrum***	-208,08	***	16.9	20.3
**Depth**	-209,73	*	2.7	23.1
